# Cardiovascular Protective Effects and Mechanisms of Action of Cacao: A Comprehensive Review

**DOI:** 10.31083/RCM45461

**Published:** 2026-02-03

**Authors:** Yu Geon Lee, Hyo-Kyoung Choi, Jin-Taek Hwang

**Affiliations:** ^1^Food Functionality Research Division, Korea Food Research Institute, 55365 Wanju-gun, Jeonbuk-do, Republic of Korea

**Keywords:** cacao, cardiovascular disease, molecular mechanism, biomarker, review

## Abstract

Cacao, the primary raw material for chocolate and certain beverages, is widely cultivated in the Americas and Asia. Furthermore, various components of cacao, including phenolic compounds, have been shown to be effective in preventing numerous diseases. Notably, cacao is particularly effective in preventing cardiovascular diseases (CVDs) by regulating various biomarkers and signaling pathways. The functionality of cacao has been reported in multiple *in vitro* and *in vivo* studies and clinical trials, thereby further confirming its efficacy. However, comprehensive reviews on the recently reported preventive effects of cacao on CVDs and the related mechanisms *in vitro*, *in vivo*, and in clinical trials remain limited. Thus, this review aimed to provide an overview of the latest research results on the effects of cacao on the prevention of CVDs and on biomarkers associated with these mechanisms. Cacao shows significant potential to prevent and mitigate CVDs, with promising findings that could shape the future of cardiovascular health and functional plant innovation. However, to fully harness the potential of cacao, future research must focus on standardizing bioactive compound content, assessing bioavailability and metabolic pathways, and establishing dose–response relationships across diverse populations.

## 1. Introduction

According to the World Health Organization, cardiovascular diseases (CVDs), including coronary artery heart disease, cerebrovascular diseases, rheumatic heart disease, and other related conditions, are the leading cause of death worldwide, with an estimated 17.9 million deaths annually [1]. CVD can be prevented in various ways, particularly for those with mild conditions or who do not require essential drug treatment. Managing risk factors through healthy eating habits and regular exercise is crucial for CVD prevention. Additionally, appropriately treating or preventing factors that can lead to CVD, such as obesity, diabetes, high cholesterol, and hypertension, is also beneficial. Biomarkers for assessing CVDs include N-terminal pro B-type natriuretic peptide (NT-proBNP), high-sensitivity C-reactive protein, and cardiac troponin (cTn), which appear in the blood when heart muscle cells are damaged [2]. Various biomarkers for CVDs have been identified, including inflammatory cytokines that swerve as useful biomarkers for heart failure and coronary artery disease owing to the close association between inflammation and CVD [2, 3]. These biomarkers play a crucial role in diagnosis and risk assessment. However, additional data are needed before these cytokines can be reliably used in clinical practice; for now, they serve primarily as reference indicators in clinical diagnosis.

The consumption of vegetables and fruits rich in antioxidants and fiber helps manage factors that contribute to CVDs [3]. Unsaturated fatty acids found in olive oil and nuts can help prevent CVDs by regulating cholesterol metabolism. Additionally, improving the diet by reducing salt intake and limiting processed foods can also help mitigate risk factors for CVD [3]. Polyphenols in vegetables, fruits, and various plants prevent CVD by reducing oxidative stress through antioxidant activity, inhibiting pro-inflammatory cytokines, and promoting vasodilation [3]. Additionally, various plant-derived components, such as dietary fiber, vitamins, fatty acids, and caffeine, have also been shown to prevent CVD [4].

The fruit of the cacao tree (*Theobroma cacao*) is the primary raw material in chocolate. *Theobroma cacao* is a tropical plant originating from South America that is widely cultivated across West Africa and Asia [5]. Cacao refers to the unprocessed state of the cacao tree, cacao pods, and cacao beans. When processed into powder, it is called cacao, and products made by mixing it with other ingredients are referred to as chocolate [5]. Cacao fruit has numerous benefits [5, 6]. For example, cacao is rich in polyphenols, which are powerful antioxidants that prevent CVDs by improving cholesterol levels and managing hypertension [6]. Additionally, cacao contains fiber and unsaturated fatty acids, which can aid in preventing CVDs [7]. However, comprehensive reviews summarizing the recently reported CVD-preventive effects of cacao and its underlying mechanisms from *in vitro*, *in vivo*, and clinical studies are currently lacking.

In this review, we aim to analyze the trends in research regarding the CVD-preventive effects of cacao and chocolate, explore their mechanisms of action in the human body, and present their potential as functional ingredients. Furthermore, this review examines how these findings can inform the development of technologies and products aligned with future trends in cacao and chocolate.

## 2. Chemical Composition of Cacao

The chemical composition of cacao varies depending on its variety and growing conditions, which directly influence its nutrient profile and potential health benefits [7]. Cacao beans are extracted from their pods and undergo fermentation and drying after harvesting [7]. Once processed, they are transported to chocolate production facilities, where they are roasted and winnowed to remove their shells. According to the Codex Alimentarius, cacao products should contain no more than 5% shell content [7, 8]. Previously, cacao pods and bean shells were considered waste, with only approximately 10% of the cacao fruit used for commercial purposes [7, 8, 9]. However, cacao bean shells (CBSs) possess a nutrient profile similar to that of cacao beans, although with a lower fat content and higher dietary fiber levels [7, 10, 11, 12]. The dietary fiber in CBS is primarily composed of pectic polysaccharides, cellulose, and hemicelluloses, which support intestinal health, help reduce cholesterol levels, and aid in regulating glucose absorption [10]. Additionally, cacao byproducts are rich in bioactive compounds, such as polyphenols, which offer various health benefits [13].

The beans of *Theobroma* species exhibit a diverse chemical composition, playing a key role in determining the nutritional value and sensory characteristics of their final products [14, 15]. Polyphenols are particularly important, contributing to the characteristic bitterness and astringency of cacao [14, 16]. Furthermore, specific chemical constituents, such as reducing sugars, free amino acids, and peptides, influence the formation of flavor compounds during post-harvest processing [17].

Cacao pods exhibit significant bioactive and antioxidant properties, likely attributable to their phytochemical composition, which closely resembles that of cacao beans [18, 19]. Their potential for incorporation into dietary applications has been widely studied, highlighting their value as a source of bioactive compounds, particularly in the plant industry [18]. Additionally, multiple studies have reported their pharmacological benefits, including antioxidant and antifungal activities [10, 20]. As a byproduct of cacao production, the husk accounts for approximately 67–76% of the total wet weight of the cacao pod and has historically been regarded as a non-consumable residue [20, 21].

CBSs comprise proteins, lipids, carbohydrates, moisture, and ash. CBSs also contain polyphenols, especially flavonoids, such as catechins and procyanidins, which provide antioxidant, anti-inflammatory, and other potential health benefits [8, 10]. The total phenolic content varies significantly depending on the extraction method and environmental factors, and the polyphenols in CBSs are sensitive to processing conditions, such as roasting and drying. CBSs also contain key methylxanthines, namely theobromine and caffeine, which are generated during cacao fermentation [22]. Theobromine, present in higher amounts than caffeine, has mild effects on the central nervous system and offers benefits, such as muscle relaxation, cardiac stimulation, and potential use, as a bronchodilator [22, 23]. Caffeine acts as a stimulant but may have adverse effects when consumed in excess [22, 24]. Furthermore, methylxanthines in CBSs enhance the bioavailability of cacao flavanols, thereby contributing to cardiovascular health. The moderate caffeine content and methylxanthines derived from CBSs enhance the value of this byproduct owing to their potential health benefits [10, 18, 24]. CBSs are also rich in minerals, particularly potassium, magnesium, calcium, and phosphorus, which are found in higher amounts in CBS than in cacao nibs owing to their accumulation in the outer shell [10]. Additionally, fermentation with specific fungi enhances mineral content. However, mineral levels vary depending on the geographic origin and soil composition where the plant was grown [10, 25]. CBSs are also a source of vitamin D, which is formed during fermentation through light activation of ergosterol. For example, CBS-fed cows produced butter with increased vitamin D levels [10, 25]. Additionally, CBSs contain notable amounts of vitamins B1 and B2, with trace amounts of vitamins B6 and D, but no detectable vitamin C. They also contain vitamin E in the form of various tocopherols, further enhancing their nutritional value [10, 11, 25]. A summary of the chemical composition of cacao beans, shells, and pods is presented in Table 1 (Ref. [10, 11, 12, 14, 15, 18, 19]).

**Table 1.** **Phytochemical composition of cacao (dry weight)**.

Sources	Category	Compounds	Contents	Ref
Bean shell	Anthocyanins	Procyanidin As	0.166 g/100 g	[10]
		Procyanidin B1	55–70 mg/100 g	[10]
		Procyanidin B2	0.0138–0.03728 g/100 g	[10]
	Flavonoids	Catechin	0.499–1.616 g/100 g	[14]
		Epicatechin	0.00022–0.084 g/100 g	[11]
		Epicatechin gallate	0.00027–0.0007 g/100 g	[11]
	Phytosterol	beta-Sitosterol	0.0078–0.211 g/100 g	[11]
		Campesterol	0.1224 g/100 g	[11]
		Cholesterol	1.712 g/100 g	[11]
		Stigmasterol	4.803–15.54 g/100 g	[11]
	Alkaloids	Caffeine	0.04–0.42 g/100 g	[10]
		Theobromine	0.39–1.83 g/100 g	[10]
		Theophylline	0.0058–0.0188 g/100 g	[10]
	Pectin		4.7–6.0 g/100 g	[12]
	Tannins		3.3–4.46 g/100 g	[12]
	Ash		5.9 g/100 g	[12]
	Total phenols		5.7 g/100 g	[12]
	Vitamins	B	3.9 µg/g	[10]
		D	0.53 µg/g	[10]
		E	1.02 µg/g	[10]
	Minerals		3.6 g/100 g	[10]
	Total dietary fiber		39.6 g/100 g	[12]
Pod	Ash		6.7–10.02 g/100 g	[18]
	Pectin		6.1–9.2 g/100 g	[18]
	Tannins		5.2 g/100 g	[19]
	Alkaloids	Theobromine	6.79 mg/100 g	[19]
	Total phenols		3.6 g/100 g	[19]
	Minerals		3.2 g/100 g	[19]
	Total dietary fiber		46.4 g/100 g	[19]
Beans	Flavonoids	Epicatechin	0.91–4.82 mg/g	[15]
		Catechin	0.07–0.62 mg/g	[15]
	Anthocyanins	Procyanidin B1	0.015–0.027 mg/g	[15]
		Procyanidin B2	0.43–2.03 mg/g	[15]
		Procyanidin B5	0.10–0.57 mg/g	[15]
		Cyanidin-3*-O*-arabinoside	1.02–1.19 mg/g	[15]
		Cyanidin-3-*O*-Galactoside	0.75–0.81 mg/g	[15]
	Alkaloids	Caffeine	0.2 g/100 g	[15]
		Theobromine	2.1–3.0 g/100 g	[15]
	Polysaccharides		12.0–16.0 g/100 g	[11]
	Ash		3.0–5.80 g/100 g	[11]
	Total phenols		4.0–8.0 g/100 g	[11]

## 3. CVDs

CVDs are characterized by the hardening or narrowing of blood vessels and the formation of blood clots. The incidence of these conditions increases due to excessive stress, irregular eating habits, and lack of exercise [1, 26]. Especially during the early stages, CVDs often remain asymptomatic or present with mild physical discomfort, contributing to underdiagnosis and delayed treatment [1, 26, 27]. However, sudden events, such as heart attacks, strokes, or heart failure, can result in immediate death. CVDs are not limited to specific age groups, sexes, or social backgrounds and are not confined solely to those of advanced age [1, 26, 27].

Regular health check-ups are particularly important for individuals with risk factors, such as hypertension, diabetes, or a family history of CVDs, as these can help in diagnosing and treating the condition [26, 27]. Methods for diagnosing CVDs include blood pressure measurement, echocardiograms, cardiovascular computed tomography scans, and electrocardiograms [26, 27]. Biochemical blood tests can also directly measure heart damage using markers, such as NT-proBNP, cTn, and creatine kinase MB (CK-MB) [2, 3, 26, 27]. NT-proBNP is secreted by myocardial cells in the ventricles in response to increased ventricular volume or overload, leading to elevated levels in the bloodstream [2, 3]. Consequently, NT-proBNP is useful for diagnosing, managing, and assessing the risk of ventricular dysfunction through blood tests [2, 3, 26, 27]. Troponin, a muscle protein complex found in striated muscle, comprises troponin C, T, and I [2, 3, 26, 27]. All troponin isoforms are present in both skeletal and cardiac muscles; however, cardiac troponin T and I are specific to the heart [2, 3]. When myocardial cells are damaged, troponin is released into the bloodstream, making blood tests crucial for diagnosing heart damage [2, 3, 26, 27]. CK-MB is an isoenzyme of creatine kinase predominantly localized in the cardiac muscle. Upon muscle damage, CK-MB is released into the bloodstream, resulting in elevated levels of this isoenzyme [2, 28]. CK-MB is commonly used to diagnose acute myocardial infarction and myocarditis.

Several other factors that may also contribute to the development of CVDs have been measured and analyzed. For instance, lipid accumulation, inflammatory responses, and oxidative stress in blood vessels can be measured to assess atherosclerotic CVD [29]. Specifically, measurements of low-density lipoprotein (LDL), high-density lipoprotein, the apolipoproteinB/A ratio, C-reactive protein (CRP), and reactive oxygen species (ROS) can help predict heart damage [29]. These biomarkers can provide an early indication of potential problems during screening, often at relatively low costs. However, accurate diagnosis requires biochemical blood analyses and additional diagnostic methods to confirm the condition.

Recent advancements in analytical technologies have led to the discovery of various metabolites, and their use in diagnostics enhances the accuracy of risk prediction [30]. Particularly, lipid-related metabolic profiles and their metabolic pathways are crucial for assessing CVD risk. Dyslipidemia and alterations in lipid metabolism are associated with abnormalities in fatty acid metabolism and mitochondrial dysfunction [30]. For example, acylcarnitine, a metabolite involved in regulating energy and fatty acid metabolism, plays an essential role in maintaining energy homeostasis in the myocardium [30]. Abnormal levels of acylcarnitine are associated with disturbances in myocardial regulation, indicating potential heart dysfunction. This highlights the importance of studying metabolic pathways and their markers in improving the precision of CVD risk assessments [30, 31].

## 4. *In Vitro* Preventive Effects of Cacao on CVDs and Their Underlying Mechanisms

The bioactive compounds of cacao offer beneficial effects on human health, particularly in relation to CVDs [6, 7, 32]. Cacao byproducts, particularly CBSs, have multiple beneficial cardiovascular effects *in vitro* [10]. Notably, CBSs bind bile salts and cholesterol during digestion, potentially reducing cholesterol absorption and contributing to improved lipid profiles [33]. Additionally, CBSs demonstrate inhibitory activity against lipase, which may lower lipid levels, indicating a beneficial hypolipidemic effect on cardiovascular health [34, 35]. CBSs also possess significant antioxidant capabilities as they are rich in polyphenolic compounds, such as catechin, epicatechin, and quercetin [36]. These polyphenols neutralize free radicals, thereby reducing oxidative stress—a critical factor in the development of CVDs—and also exhibit anti-inflammatory properties, further supporting cardiovascular health [37, 38, 39]. Additionally, CBSs contain substantial dietary fiber, which can bind cholesterol and bile acids, potentially lowering blood cholesterol and improving lipid profiles, thereby reducing cardiovascular risk [35].

Cacao pods have shown promising cardiovascular benefits through several *in vitro* mechanisms [40]. Extracts from cacao pods inhibit enzymes associated with hypertension and hyperuricemia, notably angiotensin-converting enzyme (ACE), which is crucial for blood pressure regulation, and xanthine oxidase (XO), involved in uric acid production. Furthermore, cacao extracts have demonstrated ACE inhibitory activity in endothelial and smooth muscle cell cultures, contributing to reduced angiotensin-II-mediated vasoconstriction [41]. These findings suggest potential roles in managing hypertension and reducing cardiovascular risk [42]. Rich in flavonoids and phenolic acids, cacao pods possess significant antioxidant activity, neutralizing free radicals and reducing oxidative stress associated with CVDs [43]. Furthermore, cacao pod extracts inhibit α-amylase, an enzyme crucial for carbohydrate digestion. This inhibition may help to regulate postprandial blood glucose levels, offering cardiovascular protection, which is particularly relevant for individuals with diabetes [44, 45].

Rich in polyphenols, particularly flavanols, such as catechins and procyanidins, cacao beans possess strong antioxidant activities, reducing oxidative stress associated with CVDs. Additionally, cacao polyphenols modulate inflammatory markers associated with atherosclerosis, such as interleukins and Tumor necrosis factor-alpha (TNF-α), potentially hindering disease progression by reducing inflammation [46]. A summary of the *in vitro* efficacy and mechanisms of action of cacao beans, shells, and pods is presented in Table 2 (Ref. [34, 36, 37, 38, 39, 42, 43, 44, 45, 47, 48, 49, 50, 51]).

**Table 2.** ***In vitro* efficacy and mechanisms of action of each component (bean shell, pod, and beans) of cacao (*Theobroma cacao*)**.

Sources	Primary compounds	Mechanisms	Cardiovascular benefits	Ref
Bean shell	Phenols	Binds bile salts and cholesterol; inhibits lipase	Reduces cholesterol absorption; lowers lipid levels	[34]
	Flavonoids	Free radical scavenging	Cardiovascular risk reduction	[36]
	Catechin	Free radical scavenging; anti-inflammatory properties	Cardiovascular risk reduction	[37]
	Epicatechin	Free radical scavenging; anti-inflammatory properties	Cardiovascular risk reduction	[37]
	Quercetin	Free radical scavenging; anti-inflammatory properties	Cardiovascular risk reduction	[37]
	Methylxanthines	Free radical scavenging; anti-inflammatory properties	Cardiovascular risk reduction	[38]
	Protocatechuic acid	Free radical scavenging; anti-inflammatory properties	Cardiovascular risk reduction	[38]
	Aqueous extract	Free radical scavenging; anti-inflammatory properties	Cardiovascular risk reduction	[39]
	Methanolic extracts	Free radical scavenging	Cardiovascular risk reduction	[39]
Pod	Phenols	Inhibits ACE and XO enzymes; free radical scavenging; cytoprotective effect	Reduces blood pressure; cardiovascular risk reduction	[42]
	Flavonoids	Inhibits ACE and XO enzymes; free radical scavenging; cytoprotective effect	Reduces blood pressure; cardiovascular risk reduction	[43]
	Ethanol extracts	Reduces oxidative stress; antibacterial activity; inhibits α-amylase and α-glucosidase	Reduces blood glucose; cardiovascular risk reduction	[44]
	Methanolic extracts	Free radical scavenging; antimicrobial activity; inhibits α-amylase & α-glucosidase	Reduces blood glucose; cardiovascular risk reduction	[44]
	Aqueous extract	Free radical scavenging; anti-parasitic activity; antihypertensive actions	Cardiovascular risk reduction	[45]
Beans	Flavonoids	Inhibits ACE and XO enzymes; free radical scavenging	Reduces blood pressure; cardiovascular risk reduction	[47]
	Phenols	Free radical scavenging; anti-inflammatory properties; cytoprotective effect	Cardiovascular risk reduction	[48]
	Aqueous extract	Inhibition of α-amylase, α-glucosidase, angiotensin-1 converting enzyme, and oxidative stress; antioxidant activity	Reduces blood pressure; cardiovascular risk reduction	[49]
	Peptides	ACE inhibition	Reduces blood pressure and cardiovascular risk	[50]
	Clovamide (caffeic acid derivative)	Free radical scavenging; cytoprotective effect; anti-inflammatory properties	Cardiovascular risk reduction	[51]
	Epicatechin	Free radical scavenging; anti-inflammatory properties	Cardiovascular risk reduction	[48]

ACE, angiotensin-converting enzyme; XO, xanthine oxidase.

## 5. *In Vivo* Preventive Effects of Cacao on CVDs and Their Underlying Mechanisms 

*In vivo* studies consistently show that cacao and its bioactive components—particularly polyphenols, epicatechin, procyanidins, and theobromine—provide cardioprotective effects via anti-inflammatory, antioxidant, vasodilatory, and antihypertensive pathways. In a murine ischemia-reperfusion model, phenolic-rich cacao extracts (5–25 mg/kg) reduced lipid peroxidation, nitro-oxidative stress, and inflammatory markers [interleukin 6 (IL-6), nuclear factor kappa B (NF-κB)], while upregulating AKT Serine/Threonine kinase 1 (p-Akt) and extracellular signal-regulated kinase 1/2 (p-Erk1/2 or mitogen-activated protein kinase (MAPK)) signaling [52]. Similarly, when cacao extracts were administered to experimental mice at daily doses of 5–25 mg/kg, they alleviated membrane peroxidation and nitro oxidative stress caused by myocardial injury and decreased the levels of inflammatory biomarkers IL-6 and NF-κB. They also increased the activation of cell signaling proteins p-Akt and p-Erk1/2 [52]. In another study, a flavonoid-rich cacao-carob blend was evaluated for cardioprotective properties when administered alone or in combination with metformin using a diabetic cardiomyopathy mouse model [53]. This blend improved glucose homeostasis and alleviated cardiac dysfunction, hypertrophy, and fibrosis. These effects were accompanied by inhibition of intracellular nicotinamide adenine dinucleotide phosphate (NADPH) oxidase, reduction in ROS production, and suppression of inflammatory cytokines, resulting from the regulation of sirtuin 1 and nuclear factor E2-related factor 2 (Nrf2) signaling pathways [53]. These findings suggest that the cacao-carob blend can prevent cardiac remodeling and dysfunction associated with diabetes.

In animal models of heart failure, supplementation with cacao bean polyphenols, epicatechin, or procyanidins significantly attenuated cardiac hypertrophy, improved systolic function, and downregulated pro-inflammatory gene expression. These results suggest that polyphenols, epicatechin, and procyanidins exhibit anti-remodeling and anti-inflammatory actions via modulation of cardiac signaling pathways, such as NF-κB and MAPK [54]. In systemic models of inflammation, cacao polyphenols reduced circulating levels of CRP, IL-6, and TNF-α, key markers involved in atherosclerosis and endothelial dysfunction [55]. In a rat model of isoproterenol-induced acute myocardial injury, pretreatment with cacao extract (100 mg/kg/day, orally for 2 weeks) significantly reduced serum levels of cardiac injury markers, including troponin, lactate dehydrogenase (LDH), and malondialdehyde (MDA), compared with the untreated myocardial infarction group [56]. Additional studies using cacao pod fractions and catechin-enriched extracts reported improvements in vascular function, enhanced nitric oxide (NO) production, and reduced LDL oxidation in rats and mice, indicating a lipid-lowering and vasodilatory mechanism [57]. Hypertensive models, including rats and aged rodents, demonstrated that cacao flavonoids and theobromine lowered blood pressure, suppressed ACE activity, and upregulated endothelial nitric oxide synthase (eNOS) and Nrf2 expression, which are critical for endothelial homeostasis and oxidative stress defense [58, 59]. Cacao-derived peptides also exhibit antihypertensive properties by binding directly to the catalytic domain of ACE, thereby reducing systemic blood pressure *in vivo* [50]. In pig models, cacao flavonoids attenuated atherosclerosis progression and oxidative damage, reinforcing their protective role in vascular health [60]. Cacao-derived theobromine reduced blood pressure and increased eNOS and Nrf2 expressions in aged hypertensive rats while also improving vascular function and decreasing IL-6 and TNF-α levels in mice [61, 62]. Furthermore, cacao shell extract restored vascular function and decreased inflammatory gene expression in aged hypertensive rats, offering a sustainable byproduct-based approach to preventing CVD [63]. Collectively, these *in vivo* studies reveal that cacao exerts multifaceted cardiovascular benefits through anti-inflammatory, antioxidant, vasodilatory, anti-hypertrophic, and endothelial-enhancing mechanisms. This evidence highlights the therapeutic potential of cacao in preventing and mitigating CVD through its hemodynamic modulation and anti-inflammatory/antioxidant effects. A summary of the *in vivo* efficacy and mechanisms of action of cacao is presented in Table 3 (Ref. [50, 54, 55, 57, 58, 59, 60, 61, 62, 63]).

**Table 3.** ***In vivo* efficacy and mechanisms of action of cacao (*T. cacao*)**.

Main component	Model	Cardiovascular effects	Ref
Cacao bean polyphenols	Heart failure mouse model	↓ Hypertrophy, ↑ systolic function, ↓inflammatory genes	[54]
Epicatechin	Heart failure mouse model	↓ Hypertrophy, ↑ systolic function, ↓inflammatory genes	[54]
Procyanidins	Heart failure mouse model	↓ Hypertrophy, ↑ systolic function, ↓inflammatory genes	[54]
Cacao polyphenols	Mice	↓C-reactive protein, ↓IL-6, ↓TNF-α,	[55]
Cacao pod fraction	Rats	↓LDL oxidation, ↓inflammatory genes, ↑vascular function	[57]
Catechin	Mice	↑NO production, ↓LDL oxidation	[57]
Cacao flavonoids	Hypertensive rats	↓Blood pressure	[58]
Cacao flavonoids	Pigs	↓ Atherosclerosis, oxidative stress	[60]
Theobromine	Hypertensive rats	↓ Hypertrophy, ↑ systolic function, ↓ACE, ↑NO production	[59]
Theobromine	Mice	↓inflammatory genes, ↑vascular function	[59]
Cacao peptides	Rats	↓Blood pressure, ↓ACE	[50]
Theobromine	Aged hypertensive rats	↓Blood pressure, ↑eNOS, ↑Nrf2	[61]
Theobromine	Mice	↓ IL-6, ↓ TNF-α, ↑vascular function	[62]
Cacao shell extract	Aged rats	↑vascular function, ↓inflammatory genes	[63]

IL, interleukin; TNF, tumor necrosis factor; NO, nitric oxide; LDL, low-density lipoprotein; ACE, angiotensin-converting enzyme; eNOS, endothelial nitric oxide synthase; Nrf2, nuclear factor E2-related factor 2. “↑”, upregulation or increased activity/expression; “↓”, downregulation or decreased activity/expression.

## 6. Preventive Effect of Cacao on CVD in CLinical Trials

To bridge mechanistic insights and human outcomes, this section reviews how cacao flavanols perform in diverse clinical contexts, ranging from large-scale trials to targeted interventions in high-risk populations.

### 6.1 Large-Scale Randomized Controlled Trials 

The Cocoa Supplement and Multivitamin Outcomes Study (COSMOS) trial is the most comprehensive randomized controlled study to date, enrolling 21,442 older adults (women ≥65 years, men ≥60 years) who received 500 mg/day of cocoa flavanols over a median follow-up of 3.6 years [64, 65]. Although the primary outcome—total CVD events—did not significantly differ from that of the placebo group (hazard ratio [HR]: 0.90; 95% CI [confidence intervals]: 0.78–1.02), a 27% reduction in cardiovascular mortality was observed (HR: 0.73; 95% CI: 0.54–0.98) [64]. Per-protocol analysis further indicated greater benefits in participants with high adherence (HR: 0.85) [64]. A secondary analysis focused on the incidence of type 2 diabetes reported no overall effect (HR: 1.04); however, a potential benefit was observed in current smokers (HR: 0.55; *p* = 0.07) [66]. Similarly, the Prostate, Lung, Colorectal and Ovarian Cancer Screening Trial (n = 91,891) found that higher chocolate intake (>2 servings/week) was associated with reduced all-cause (HR: 0.84–0.89), CVD (HR: 0.76–0.79), and Alzheimer’s disease mortality (HR: 0.69), particularly in never-smokers [67]. No significant associations were observed for mortality due to cancer, cerebrovascular, or respiratory diseases.

### 6.2 Trials in Pre- and Post-Menopausal Women 

Two 6-month randomized controlled trials in Spain assessed the effects of 10 g/day intake of 99% cocoa chocolate. One study (n = 132) reported reductions in body fat mass (–0.63 kg) and body fat percentage (–0.79%) without significant changes in body weight, body mass index, or insulin sensitivity [68]. Another study (n = 140) found no overall effects on arterial stiffness or blood pressure, although pulse pressure (–2.05 mmHg) and systolic blood pressure (–4.64 mmHg) significantly decreased in the overweight/obese subgroups [69]. In contrast, a 4-week trial in 32 overweight/obese pre-menopausal women administered 1.2 g/day of cocoa flavanols revealed no improvements in insulin resistance, glucose uptake, or body composition [70].

### 6.3 Platelet Function and Endothelial Effects

The ECLAIR trial, an open-label pilot study conducted in Trinidad and Tobago, administered 30 g/d of 65% dark chocolate to 20 patients with coronary artery disease receiving dual antiplatelet therapy [71]. They observed an 11.9% reduction in platelet reactivity units, enhancing the effect of clopidogrel, with no notable change in response to aspirin. A Turkish crossover study involving patients with heart failure found that both milk and dark chocolate reduced NT-proBNP levels [72]. However, only dark chocolate significantly improved flow-mediated dilation (FMD) (from 8.9% to 14.0%).

### 6.4 Peripheral Arterial Disease (PAD) and Inflammation 

In the COCOA-PAD trial, patients with PAD who consumed 15 g/day of cocoa flavanols for 6 months showed improved walking distance, along with upregulation of antioxidant and mitochondrial markers, such as heme oxygenase-1, NAD(P)H: quinone oxidoreductase 1, ubiquinol cytochrome c reductase, and phosphorylated Nrf2 [73]. These findings suggest restored mitochondrial function and reduced oxidative stress. Similarly, among patients undergoing hemodialysis in Brazil, intake of 40 g of 70% dark chocolate thrice weekly over 2 months significantly reduced TNF-α levels without affecting potassium or phosphorus levels [74].

### 6.5 Acute Vascular Responses 

A UK crossover study reported that 1350 mg of cocoa flavanols acutely improved FMD in both femoral and brachial arteries (+2.9% and +3.0%, respectively), reduced systolic blood pressure (–7.2 mmHg), and increased microvascular diameter in the foot within 2 h [75]. These effects were more pronounced in individuals with baseline endothelial dysfunction. Conversely, a Belgian study using 790 mg of cocoa flavanols observed no significant acute vascular improvements in individuals with type 2 diabetes [76]. Modest postprandial benefits in FMD and diastolic blood pressure were noted only in non-diabetic participants, regardless of treatment.

### 6.6 Null or Inconclusive Findings

Despite overall positive trends, several trials report null or mixed outcomes. A cognitive substudy of the COSMOS trial (COSMOS-Clinic) reported no significant effects of 500 mg/day cocoa flavanol supplementation on global cognition, episodic memory, or executive function; however, a modest improvement in executive function was noted among participants with low baseline diet quality [77]. Similarly, the Pocket-4-Life study found that consuming cocoa-based confectionery combined with espresso for 1 month did not significantly alter cardiovascular or metabolic biomarkers, despite increased saturated fat and sugar intake [78]. In a 4-week placebo-controlled randomized controlled trial involving healthy adults, supplementation with cocoa flavanols, epicatechin, methylxanthines, or their combination yielded no meaningful effects on vascular function or lipid profiles [79]. Additionally, an ancillary analysis of the COSMOS trial examining age-related macular degeneration (AMD) found no significant reduction in AMD risk with cocoa flavanol intake over a median follow-up of 3.6 years (HR: 0.87; 95% CI: 0.71–1.08; *p* = 0.21). However, a modest early protective effect was suggested during the first 2 years (HR: 0.77; 95% CI: 0.59–1.01), highlighting the need for further investigation into potential time-dependent vascular mechanisms [80]. These findings, summarized in Table 4 (Ref. [64, 65, 66, 67, 68, 69, 70, 71, 72, 73, 74, 75, 76, 77, 78, 79, 80]) highlight considerable variability in clinical outcomes depending on population characteristics, dietary background, dose, and intervention duration. While the overall evidence supports the vascular and metabolic benefits of cacao, inconsistencies in trial design and reporting standards warrant cautious interpretation and underscore the need for further targeted, biomarker-driven studies.

**Table 4.** **Cardioprotective effects of cacao (*T. cacao*) in humans according to clinical trials and meta-analyses**.

PMID	Year	Country/region	Study type/name	Follow-up period	Study size	Supplemented	Findings	Ref
35294962	2022	United States	Randomized, double-blind, placebo-controlled trial (COSMOS: Cocoa Supplement and Multivitamin Outcomes Study)	Median 3.6 years (IQR 3.2–4.2 years)	21,442 healthy older adults (12,666 women ≥65 years, 8776 men ≥60 years)	Cocoa extract capsules, 500 mg cocoa flavanols/day (including 80 mg (–)-epicatechin)	Primary outcome: No significant reduction in total CVD events (HR: 0.90; 95% CI: 0.78–1.02; *p* = 0.11). Secondary outcome: 27% reduction in CVD death (HR: 0.73; 95% CI: 0.54–0.98; *p* = 0.04). Per-protocol analysis: HR 0.85 (95% CI: 0.72–0.99), suggesting benefit with adherence. No safety concerns.	[64]
32746952	2021	Spain	Randomized controlled, parallel trial	6 months	132 post-menopausal women (67 IG, 61 CG)	10 g/day of 99% cocoa chocolate (65.4 mg polyphenols, 26.1 mg epicatechin)	↓ Body fat mass (–0.63 kg, *p* = 0.019), ↓ body fat % (–0.79%, *p* = 0.004). No changes in body weight, BMI, insulin, HOMA-IR, or SBP. Reductions in fat were observed across the trunk, arms, and legs. No adverse effects were reported.	[68]
36100318	2022	Trinidad and Tobago	Prospective, open-label pilot clinical trial/Effect of Cocoa on Platelet Function (ECLAIR)	1 week	20 patients with stable coronary artery disease on dual antiplatelet therapy (ASA + clopidogrel)	30 g/day of 65% cocoa dark chocolate (3 × 10 g/day with meals)	↓ PRU by 26.85 units (11.9% relative reduction; *p* = 0.001) → significantly enhanced clopidogrel effect. No significant effect on aspirin response (ARU: ↓17.65, *p* = 0.351). No adverse events.	[71]
37816167	2023	United States	Randomized, double-blind, placebo-controlled trial/COSMOS (T2D secondary outcome analysis)	Median 3.5 years (IQR: 3.3–4.2)	18,381 participants (12,666 women ≥65 years, 8776 men ≥60 years; all without diabetes at baseline)	500 mg/d cocoa flavanols (including 80 mg (–)-epicatechin) via capsules	No significant effect on incident T2DM (HR: 1.04, 95% CI: 0.91–1.20, *p* = 0.58). Null effect consistent across sex, BMI, diet, activity, and multivitamin use. Possible trend toward benefit in current smokers (HR 0.55, *p* = 0.07).	[66]
34329196	2021	United States	Prospective cohort analysis/PLCO cancer screening trial (post hoc)	Mean 13.5 years (SD 3.3)	91,891 adults (aged 55–74 years)	Chocolate intake (0 to >2 servings/week; 1 serving ≈ 28.35 g) assessed by FFQ.	Chocolate intake was associated with lower all-cause (HR: 0.84–0.89), cardiovascular (HR: 0.76–0.79), and Alzheimer’s disease mortality (HR: 0.69 for >2 servings/week). No effect on cancer, cerebrovascular, or respiratory mortality. Stronger effects in never-smokers. Nonlinear dose-response observed.	[67]
35288332	2022	United States	Randomized, double-blind, placebo-controlled trial/COSMOS (design & baseline)	Planned median: 3.6 years	21,442 adults (12,666 women ≥65 years; 8776 men ≥60 years)	500 mg/day cocoa flavanols (including 80 mg (–)-epicatechin) vs placebo	No clinical outcome yet. Design: 2 × 2 factorial trial testing cocoa extract and multivitamins for prevention of CVD and cancer. Successful randomization, high compliance, and broad representativeness. Detailed biospecimen and clinic substudy frameworks were provided.	[65]
38189132	2024	United States	Phase II RCT with mechanistic sub-study/COCOA-PAD trial	6 months	44 patients with PAD (16 with muscle biopsy)	15 g cocoa/day including 75 mg (–)-epicatechin	Cocoa flavanols improved walking distance and reduced central nuclei in type II fibers. Associated with ↑ heme oxygenase-1/NAD(P)H:quinone oxidoreductase 1, ↑ ubiquinol cytochrome c reductase (complex III), and ↑ Nrf2 phosphorylation. epicatechin restored mitochondrial function and reduced ROS in PAD myotube models; effects were reversed by Nrf2 inhibition.	[73]
38070683	2023	United States	RCT, in-person cognitive substudy/COSMOS-Clinic	2 years	573 older adults (mean age 69.6 years)	500 mg/day cocoa flavanols, including 80 mg (–)-epicatechin	No significant effects on global cognition (Δ = –0.01 SU), episodic memory (Δ = –0.01 SU), or executive function (Δ = 0.003 SU). Subgroup analysis suggests a benefit in participants with lower diet quality at baseline (executive function Δ = 0.13 SU).	[77]
36164983	2022	United Kingdom	Randomized, double-blind, placebo-controlled crossover trial	Acute (2 h post-intervention)	22 adults (11 healthy, 11 with T2DM)	1350 mg/day cocoa flavanols, including 255 mg (–)-epicatechin	Acute CF intake ↑ FMD in femoral and brachial arteries (Δ +2.9% and +3.0%); ↑ microvascular diameter in the foot (+3.5 µm); ↓ SBP (–7.2 mmHg) and ↓ PWV (–1.3 m/s). Greater endothelial dysfunction at baseline in T2DM, particularly in FA.	[75]
36657913	2023	Brazil	Open-label, controlled clinical trial	2 months	46 patients with chronic kidney disease undergoing hemodialysis (35 chocolate, 11 control)	40 g dark chocolate (70% cocoa) 3×/week during HD	↓ TNF-α levels (*p* = 0.008). No change in IL-6, malondialdehyde, oxLDL, potassium, or phosphorus. Chocolate was well-tolerated, without adverse metabolic effects.	[74]
35860885	2022	Turkey	Randomized, crossover clinical trial	2 weeks per phase	20 patients with heart failure and reduced ejection fraction	Milk and dark chocolate (unspecified dose)	↓ NT-proBNP after both chocolates. ↑ FMD after dark chocolate only (8.9% → 14.0%, *p* = 0.019). Suggests endothelial function improvement and acute cardiac biomarker benefit.	[72]
36771271	2023	United Kingdom	RCT, double-blind, placebo-controlled/Cocoa-IR Study	4 weeks	32 overweight/obese pre-menopausal women	1.2 g/day cocoa flavanols (2 × 609 mg), 190 mg (–)-epicatechin	No significant improvement in insulin resistance (HOMA-IR) or insulin-stimulated glucose uptake (M-value). No effect on body weight or composition. No changes in fat/carbohydrate oxidation. Effect size was minimal.	[70]
32545478	2020	Spain	Randomized controlled parallel trial	6 months	140 post-menopausal women (71 IG, 66 CG)	10 g/day of 99% cocoa chocolate (65.4 mg polyphenols, 26.1 mg epicatechin)	No effect on SBP/DBP/arterial stiffness overall. ↓ Pulse pressure (PP) in total IG (–2.05 mmHg, *p* = 0.048). Significant ↓ SBP (–4.64 mmHg, *p* = 0.020) and ↓ PP (–3.88 mmHg, *p* = 0.003) in the overweight/obese subgroup only. No changes in glucose, insulin, lipids, or HOMA-IR.	[69]
35807872	2022	Belgium	Randomized, double-blind, placebo-controlled crossover trial	Acute (70 min post-ingestion)	35 total (24 non-diabetic, 11 with T2DM)	790 mg cocoa flavanols including 149 mg (–)-epicatechin (capsule)	No acute CF effect on FMD, BP, or muscle microvascular reactivity. Postprandial changes (↑ FMD and ↓ DBP) were observed only in the non-DM group, regardless of CF/placebo. Suggests limited acute CF vascular effects in T2DM.	[76]
32728879	2020	Italy	Randomized, double-blind, three-arm crossover RCT/Pocket-4-Life	1 month per intervention arm	21 healthy volunteers	(1) 1 cup/day espresso (2) 3 cups/day espresso (3) 1 cup/day espresso + 2 cocoa-based confectionery/day	No significant changes in BP, glucose, insulin, lipids, HOMA-IR, NO, IL-8, VEGF, TNF-α, or TMAO in any group. Cocoa confectionery intake increased saturated fat and sugar but had no cardiometabolic impact.	[78]
40146119	2025	United States	Randomized, double-blind, placebo-controlled trial/COSMOS (AMD ancillary study)	Median 3.6 years	21,442 (subset for AMD)	500 mg/day cocoa flavanols incl. 80 mg (–)-epicatechin	No significant effect on AMD risk overall (HR: 0.87; *p* = 0.21); possible early benefit in first 2 years (HR: 0.77; 95% CI: 0.59–1.01)	[80]
39901369	2024	Germany	Randomized, placebo-controlled trial	4 weeks	75 healthy adults (five groups, n = 15 each)	Encapsulated flavanol-rich cocoa, epicatechin (EC), methylxanthines (MX), EC + MX, or placebo	No significant changes in PWV, BP, lipids, or endothelin-1 in any group. No vascular or lipid benefits observed in healthy participants.	[79]

AMD, age-related macular degeneration; PMID, PubMed Identifier; RCT, randomized controlled trial; ASA, aspirin; PAD, peripheral arterial disease; T2DM, type 2 diabetes mellitus; y, years; FMD, flow-mediated dilation; PRU, platelet reactivity units; BP, blood pressure; DBP, diastolic blood pressure; SBP, systolic blood pressure; BMI, body mass index; HOMA-IR, homeostasis model assessment-estimated insulin resistance; VEGF, vascular endothelial growth factor; TMAO, trimethylamine N-oxide; IG, intervention group; CG, control group; SU, standard units; ROS, reactive oxygen species; CVD, cardiovascular disease; CF, cocoa flavanols; PWV, pulse wave velocity; NT-proBNP, N-terminal pro B-type natriuretic peptide. “↑”, upregulation or increased activity/expression; “↓”, downregulation or decreased activity/expression.

## 7. Heterogeneity Analysis of Results

Although numerous clinical studies suggest cardiovascular benefits of cacao, the results remain inconsistent owing to substantial heterogeneity across study designs and populations. Variations in flavanol dosage, intervention duration, and cacao product formulations (e.g., extract, powder, and chocolate) contribute to divergent outcomes. For instance, short-term trials may fail to capture sustained physiological changes, whereas long-term studies often reveal more robust effects on vascular function and inflammatory markers. Differences in baseline health status—such as age, menopausal stage, obesity, or cardiometabolic disease—also influence individual responsiveness. Furthermore, inter-individual differences in gut microbiota composition and genetic polymorphisms affecting flavanol metabolism (e.g., catechol-O-methyltransferase (COMT), eNOS) can modify bioavailability and efficacy. In addition, the selection of endpoints (e.g., FMD, homeostasis model assessment-estimated insulin resistance, NT-proBNP, and cytokines) and analytical methods varies across trials, limiting direct comparability. These factors highlight the need for standardized intervention protocols, biomarker-driven stratification, and personalized approaches in future cacao-related clinical research.

## 8. Limitations

Despite the comprehensive integration of *in vitro*, *in vivo*, and clinical evidence in this review, certain limitations should be acknowledged to improve clarity and future research planning.

### 8.1 Study Design Limitations

Studies exhibited considerable heterogeneity in terms of experimental design, flavanol dosage, intervention duration, and forms of cacao products used (e.g., cocoa powder, dark chocolate, and extracts). This variation limits direct comparability and hinders meta-analytic synthesis. In many cases, flavanol content is not standardized, making dose–response interpretations difficult.

### 8.2 Mechanism Research Limitations

Although many mechanistic insights are derived from *in vitro* and animal models, these often lack consistency in experimental protocols and do not fully replicate the complexity of human cardiovascular pathophysiology. The roles of individual bioactive compounds, such as (–)-epicatechin, theobromine, or fiber, are not always isolated in mechanistic studies, and interactions among components remain underexplored.

### 8.3 Clinical Translation Limitations

Long-term, well-controlled clinical trials with standardized flavanol content and biomarker-guided endpoints also remain sparse. Most studies do not account for individual variability in responses, which may arise from differences in gut microbiota composition, dietary background, and genetic polymorphisms. This presents a major barrier to precision nutrition applications and the clinical adoption of cacao-based interventions. These limitations highlight the necessity for harmonized methodologies, personalized approaches, and robust clinical designs to fully validate the preventive role of cacao in cardiovascular health.

Despite the noted limitations, current evidence suggests that cacao flavanols may offer selective cardiovascular benefits, particularly in populations with elevated cardiometabolic risk. Evidence supports improvements in endothelial function, platelet inhibition, and systemic inflammation; however, results for total CVD event reduction remain inconsistent. Critical factors, such as dosage, adherence, baseline health status, and intervention duration, appear to influence clinical outcomes.

## 9. Conclusions

**Fig. 1.**
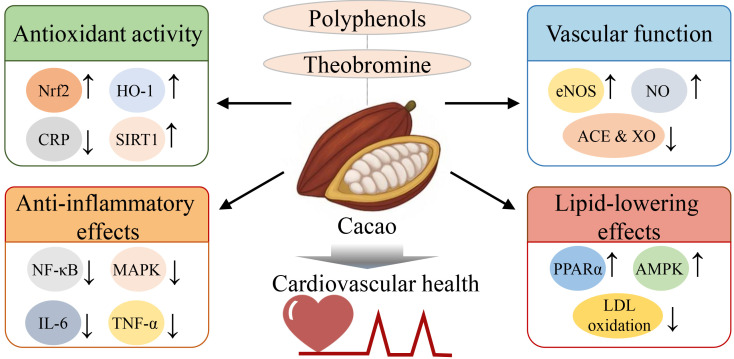
**Proposed mechanisms underlying the cardiovascular-protective effects of cacao bioactives**. This figure highlights the key bioactive components of cacao—particularly polyphenols and theobromine—and their associated molecular mechanisms contributing to cardiovascular protection. Cacao enhances antioxidant defenses by upregulating Nrf2, heme oxygenase-1 (HO-1), and sirtuin 1 (SIRT1), while reducing circulating levels of C-reactive protein (CRP). It exerts anti-inflammatory effects by downregulating NF-κB, MAPK, IL-6, and TNF-α. In terms of vascular function, cacao promotes eNOS expression and NO production, while suppressing ACE and XO, thereby supporting vascular homeostasis. For lipid-lowering effects, cacao activates peroxisome proliferator activated receptor alpha (PPARα) and AMP-activated protein kinase (AMPK) pathways and inhibits low-density lipoprotein (LDL) oxidation, contributing to improved lipid metabolism. These molecular actions highlight the multifaceted role of cacao in mitigating cardiovascular disease risk by modulating oxidative stress, inflammation, endothelial dysfunction, and lipid dysregulation. “↑”, upregulation or increased activity/expression; “↓”, downregulation or decreased activity/expression. MAPK, mitogen-activated protein kinase; AMP, adenosine monophosphate.

Cacao and its byproducts, particularly CBSs, exert multifaceted cardioprotective effects via antioxidant, anti-inflammatory, endothelial-modulating, and lipid-regulating mechanisms. These effects are mediated through key molecular targets such as NF-κB, Nrf2, eNOS, and ACE, contributing to improved vascular function, reduced oxidative stress, and metabolic homeostasis (Fig. 1). However, heterogeneity in cacao dosage, intervention duration, product formulation, and participant characteristics has led to inconsistent clinical outcomes. While some trials demonstrate significant improvements in vascular biomarkers, others report null effects, particularly in metabolically compromised populations or at sub-therapeutic flavanol doses. These inconsistencies emphasize the need for standardized intervention protocols, biomarker-guided endpoints, and personalized nutrition strategies. This review integrates mechanistic and clinical evidence, highlighting converging molecular pathways and emerging bioresources such as CBSs. It also identifies key knowledge gaps, including insufficient long-term data, limited understanding of host–microbiome interactions, and interindividual variability in response. To advance this field, future research should focus on: (1) conducting stratified randomized controlled trials in high-risk populations (e.g., PAD, heart failure, metabolic syndrome); (2) standardizing flavanol dose, form, and duration across studies; (3) integrating gut microbiome and pharmacogenomic profiling to understand variability in response; and (4) evaluating long-term effects on validated CVD biomarkers such as NT-proBNP, FMD, and TNF-α.

## Abbreviations

ACE, angiotensin-converting enzyme; CBS, cacao bean shells; CVD, cardiovascular disease; CRP, C-reactive protein; cTn, cardiac troponin; FMD, flow mediated dilation; HDL, high-density lipoprotein; LDL, low-density lipoprotein; NT-proBNP, N-terminal pro B-type natriuretic peptide; PAD, peripheral artery disease.
